# Myocilin variations and familial glaucoma in Taxiarchis, a small Greek village

**Published:** 2008-04-25

**Authors:** Mary K. Wirtz, Anastasios G. P. Konstas, John R. Samples, Kostantinos Kaltsos, Athanasios Economou, Antonios Dimopoulos, Irene Georgiadou, Michael B. Petersen

**Affiliations:** 1 Casey Eye Institute, Department of Ophthalmology, Oregon Health & Sciences University, Portland, OR; 2 Glaucoma Unit, 1^st^ University Department of Ophthalmology, AHEPA Hospital, Thessaloniki, Greece; 3 Department of Genetics, Institute of Child Health, Athens, Greece

## Abstract

**Purpose:**

To initiate a prospective study of glaucoma in a Greek village reported over 30 years ago to have several large families with primary open-angle glaucoma (POAG).

**Methods:**

A random group of 126 villagers from Taxiarchis, Greece was examined in the village community center. The detailed evaluation included ophthalmic and general history, measurement of blood pressure, intraocular pressure (IOP), and central corneal thickness (CCT) as well as evaluation of the optic nerve status.

**Results:**

The incidence of glaucoma approached 18% in this small isolated village. Myocilin variants were present in almost half of the individuals screened with Arg76Lys and Thr377Met being the most common finding (25% and 17%, respectively). Over half of the individuals with the Thr377Met mutation were diagnosed with glaucoma. Two of these patients were homozygous for the Thr377Met mutation. Three individuals with the Arg76Lys polymorphism had glaucoma; however, two of these individuals also had the Thr377Met mutation. Only two patients with pseudoexfoliation were identified.

**Conclusions:**

The incidence of glaucoma and the Thr377Met *MYOC* mutation in this population is much higher than that reported for other European populations.

## Introduction

Glaucoma is a common blinding disease that was originally described in Greece around 500 BC. In ancient Greece, Aristotle and Hippocrates treated patients with “glaucosis,” associated with the gray-green color of the pupil in the last stages of glaucoma [1]. Today, glaucoma is recognized as a group of eye diseases resulting from optic nerve damage. Primary open-angle glaucoma (POAG) is the most common form of glaucoma in the United States, Europe, and Australia comprising 75%–95% of the glaucoma cases [2]. Population-based studies of POAG in Europe have reported prevalence rates ranging from 1.1% in the Rotterdam Study [3] to 3.8% in the Thessaloniki Eye Study [4].

Exfoliative glaucoma (XFG) is a secondary glaucoma that is found in high frequency in certain regions of Europe. This disease is characterized by flakes of granular material at the pupillary margin of the iris and throughout the inner surface of the anterior chamber. The prevalence of XFG is extremely high in the Scandinavian countries and in Greece [4-7]. Recently, an association of a single nucleotide polymorphism (SNP) in the *LOXL1* gene with exfoliation was identified in the Icelandic population [8].

Taxiarchis, a small village in northern Greece with 1,100 inhabitants, was considered to have a surprisingly high incidence of glaucoma [9]. This isolated population located on Mount Holomondas appeared to be enriched for familial glaucoma. In the only previous attempt to investigate the problem, Georgiades et al. [9] examined a cohort of the villagers and identified 20 individuals with glaucoma. Fourteen of the individuals were from one large family comprised of five generations with a clear autosomal dominant inheritance pattern of POAG. Only two of the 20 individuals with glaucoma were sporadic cases.

Mutations in *myocilin* (*MYOC*), the first POAG gene to be identified, have been found in approximately 4% of POAG patients (Myocilin allele-specific phenotype database) [10]. The Thr377Met *MYOC* mutation has been identified in several ethnic groups from Greece, Britain, Finland, India, Australia and the United States [11-17]. Distinct haplotypes are associated with the Thr377Met *MYOC* mutation, suggesting that this mutation has arisen independently at least three times [11]. To date, all individuals of Greek ancestry with the Thr377Met *MYOC* mutation share the same haplotype, suggesting a common founder for these families.

The clinical phenotype of the Thr377Met *MYOC* variant is intermediate between the more aggressive and severe glaucoma found in patients with the Pro370Leu mutation and the mild presentation of the Glu368Stop mutation. The age of onset in patients with the Thr377Met ranges from 14 to 66 years [12,17].

The Arg76Lys variation in *MYOC* appears to be a non-disease-causing single nucleotide polymorphism (SNP; at the Myocilin allele-specific phenotype database) [18,19]. The designation of this SNP as not being a factor in POAG is based upon the similar frequency of the alleles in control and patient populations as well as the conservation of charge of the arginine and lysine amino acids. In addition, the Arg76Lys change has been reported to segregate with the non-disease haplotype in a POAG family [20]. Thus, the Arg76Lys variant is considered to be a normal non-disease-causing SNP.

Based upon the high reported incidence of glaucoma in Taxiarchis and our previous findings of the Thr377Met *MYOC* mutation in the Greek population [11], we undertook a community-based study to determine if this variant was present in this village.

## Methods

A random group of 126 villagers in Taxiarchis was interviewed and examined after obtaining informed consent following the protocol approved by the Bioethics Committee of the Medical School of Aristotle University of Thessaloniki. The principles of the Declaration of Helsinki were followed. The interview included familial and personal histories of glaucoma. The detailed evaluation at the community center in Taxiarchis included ophthalmic and general history, measurement of blood pressure, intraocular pressure (IOP), central corneal thickness (CCT), and evaluation of the optic nerve status. Slit-lamp biomicroscopy was performed in selected cases. IOP was measured with two recently calibrated Reichert TONO-PEN^®^ XL Applanation Tonometers (Reichert Ophthalmic Instruments, Depew, NY). Optic nerve cupping was evaluated using a direct ophthalmoscope in an undilated state. Pachymetry with an ultrasound Pachmate DGH55 instrument (DGH Technology, Exton, PA) was used to determine the CCT for each eye. The purpose of the on-site clinical screening was not to make a definitive diagnosis but rather to decide who to refer for further investigation based upon high IOPs and/or suspicious optic nerve appearance. Glaucoma classification was based upon characteristic glaucomatous optic nerve findings such as cup to disk ratios of 0.6 or greater (a cup to disk ratio of 0.6 is defined as glaucomatous in Greece [21]) in one or both eyes, asymmetry of cup to disk ratios of 0.2 or greater, and erosion or notching of rims. Individuals were classified as glaucoma suspects whenever characteristics such as violation of the inferior, superior, nasal, and temporal (ISNT) rule, asymmetry in cupping, a focal notch in the nerve, or IOP greater than 21 mmHg were present [22]. In our initial screen, the diagnosis of the glaucoma suspect was a means of referring the patient for more definitive study. Venous blood was obtained for DNA extraction using the standard salting out methods.

Following the field visit, villagers with a high IOP and the appearance of glaucomatous disc damage were given a comprehensive exam at the Glaucoma Unit of the 1^st^ University, Department of Ophthalmology, American Hellenic Educational Progressive Association (AHEPA) Hospital, Thessaloniki, Greece. Patients underwent best corrected distance Snellen visual acuity assessment, corneal pachymetry, a reliable visual field (<30% fixation losses, false positives and negatives) with Humphrey 24–2, gonioscopy-detailed assessment of anterior and posterior segments before and after dilatation of the pupil, and a scanning laser polarimetry with variable corneal compensation (GDX VCC) exam when possible. Two separate IOP measurements were conducted at this visit between 11:00 AM and 1:00 PM All patients underwent a detailed assessment for the presence of exfoliation after pupillary dilation. Several exfoliation-related clinical signs were recorded in an attempt to create a “phenotype score.”

Following the comprehensive hospital exam, patients were divided into four groups-glaucoma, suspicion for glaucoma, ocular hypertensive (OHT), and normal. The definition of glaucoma in this investigation adhered to the AAO Preferred Practice Patters criteria-at least one IOP measurement of 22 mmHg or more (if the IOP was never greater that 22 mmHg, it was considered low tension glaucoma), reproducible visual field damage, pathologic optic nerve cupping, violation of the ISNT rule, and cup-to-disc ratio of 0.6 or greater. During the comprehensive exam, disc size was measured with a Volk stereoscopic lens and taken into consideration.

**Figure 1:**
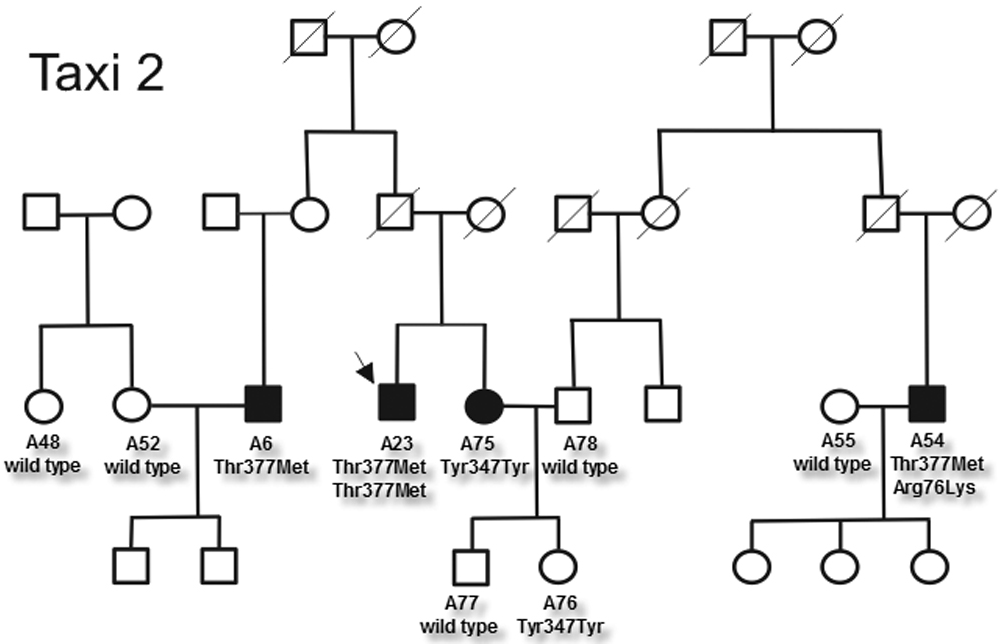
Segregation of primary open-angle glaucoma in a large family from Taxiarchis (Taxi 2). Note that A23 is homozygous for the Thr377Met mutation. A filled-in symbol indicates that the person has been diagnosed with POAG. The number under the symbol indicates the specific code given to the family member who participated in the study. The *MYOC* genotype is denoted below the code. The arrow indicates the proband for the pedigree.

### *Myocilin* screening

The promoter region and exons 1–3 of *MYOC* were sequenced at the Portland Veterans Administration Medical Center (Portland, OR) as previously described [23]. Microsatellite marker analysis in the GLC1A region including D1S452, D1S1619, D1S2790, and D1S242 was performed at deCODE (Reykjavik, Iceland)

## Results

One hundred and twenty-six villagers in Taxiarchis were examined for glaucoma. The age of exam represents the age of diagnosis for those individuals with glaucoma since this was the first time that they had been examined. The average age of the people in the study was 60 years and ranged from 8 to 90 years old. Twenty-one of the 126 villagers examined (16%) were diagnosed as having glaucoma, and 10 of the villagers (7%) examined were diagnosed as glaucoma suspects. The highest recorded IOP was 65 and 36 mmHg in the right and left eye, respectively. Family trees were constructed based upon the information given by the participants. Seventeen of the villagers diagnosed with glaucoma were from five families with at least two or more affected individuals. The remaining four glaucoma patients were isolated cases.

During the subsequent comprehensive exam at the Department of Ophthalmology of 1^st^ University in Thessaloniki, Greece, 34 of the villagers were reexamined including 20 of the 21 villagers with glaucoma. The diagnosis of POAG was confirmed in all 20 of these individuals. The classification of two of the suspects was changed to POAG without knowledge of their *MYOC* genotype. Twenty-two villagers (17%) were confirmed as having glaucoma, six (4%) were classified as glaucoma suspects, and three (2%) were reclassified as ocular hypertensives. Only 2 of the 23 individuals with glaucoma had XFG. Two of the patients (A6 OS and A15 OD) had trabeculectomies with mitomycin C after the study visit. Patient A9 had bilateral insertion of Glaukos stents.

**Figure 2:**
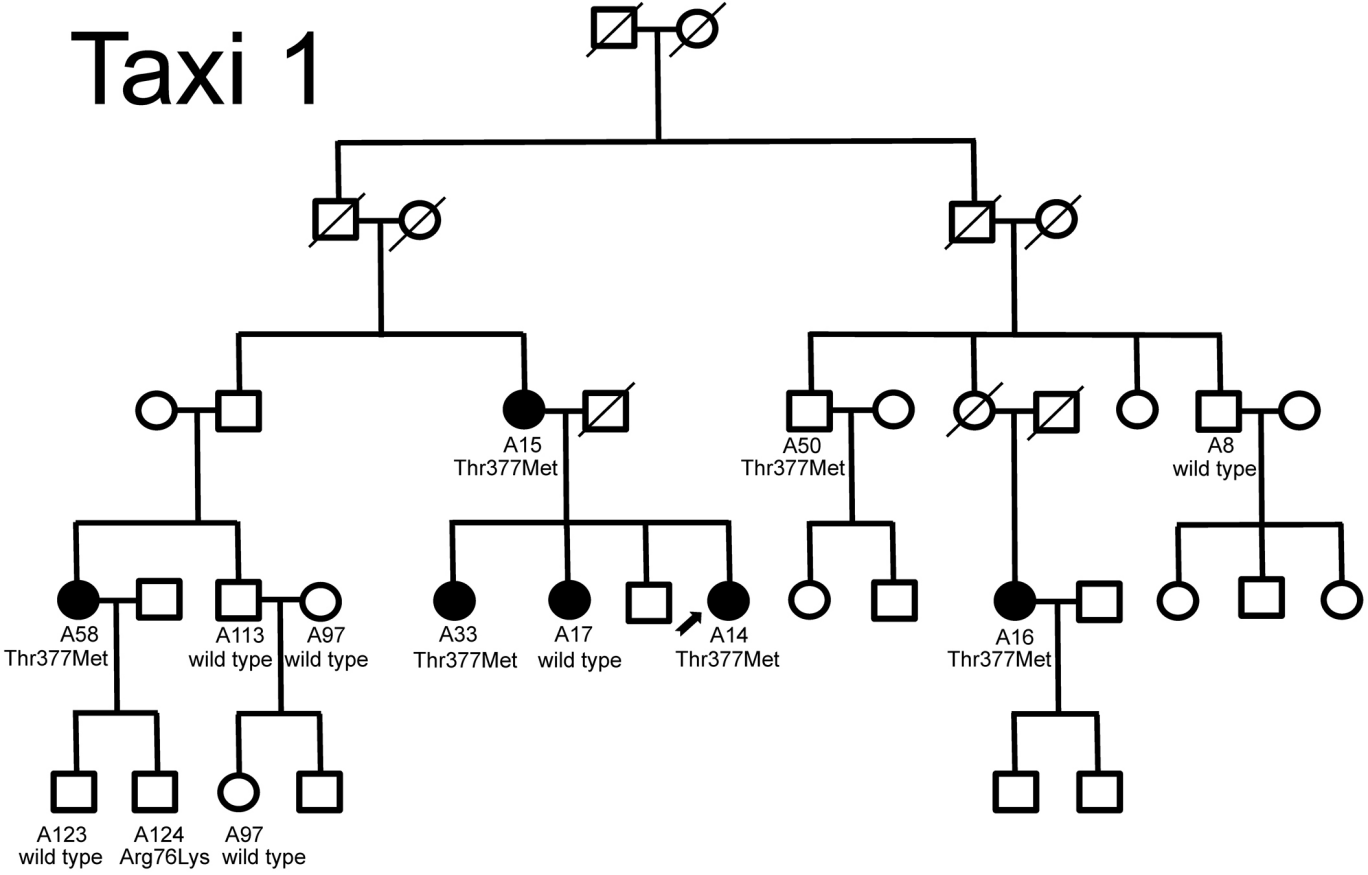
. Segregation of primary open-angle glaucoma and the Thr377Met *MYOC* mutation in a large family from Taxiarchis (Taxi 1). A filled-in symbol indicates that the person has been diagnosed with POAG. The number under the symbol indicates the specific code given to the family member who participated in the study. The *MYOC* genotype is denoted below the code. The arrow indicates the proband for the pedigree.

### *Myocilin* genotype

Sequencing of the three exons and promoter of *MYOC* in 124 of the villagers identified four *MYOC* variants, Thr377Met, Arg76Lys, Tyr347Tyr, and Thr438Thr (see Table 1). The Arg76Lys was the most common with its presence in 31 people; 21 individuals possessed the Thr377Met variant; seven had the Tyr347Tyr; and one individual had the Thr438Thr polymorphism. Of the 124 villagers screened, 55 villagers possessed one or more of the above *MYOC* variants (see Table 1).

**Table 1:** Summary of *MYOC* variants and clinical presentation in villagers.

**Genotype**	**Number of villagers with phenotype**						
	**POAG**	Glaucoma*	**Exfoliation**	**OHT**	**Suspect**	**Normal**	**Total**
Thr377Met	9			1		6	16
Thr377Met/Thr377Met	2						2
Thr377Met/Arg76Lys	2				1		3
Arg76Lys			1	1		25	27
Arg76Lys/Thr438Thr						1	1
Tyr347Tyr			1	1		4	6
Wild Type	8	1			4	56	69
Not Genotyped					1	1	2
Total	21	1	2	3	6	93	126

Summary of *MYOC* variants and ocular findings in 126 villagers from Taxiarchis, Greece. POAG indicates primary open angle glaucoma; OHT indicates ocular hypertension. The asterisk indicates that the patient has not had a comprehensive examination to rule out exfoliation glaucoma.

Of the 21 individuals with the Thr377Met variant, 13 were diagnosed with POAG and two had OHT. One person with normal IOP and cup to disc ratios had a visual field consistent with POAG and was classified as a suspect (see Table 2). Two individuals (A23 and A126) were homozygous for the Thr377Met mutation, and both had POAG. A23 had the highest recorded IOP (65 mmHg OD). At the comprehensive reexamination at the 1^st^ University Department of Ophthalmology in Thessaloniki, Greece, the IOP in his right eye was still high at 60 mmHg. Both of his parents were deceased. His sister who also had glaucoma did not have the Thr377Met variant (see Figure 1). The other villager who was homozygous for the Thr377Met mutation (A126) was 16 years old at the time of examination; she had pressures of 25 OU and asymmetric cup to disc ratios with visual field loss in the right eye. Her mother was 51 years old at the time of examination and had normal pressures (17 mmHg OU) with cup to disc ratios of 0.3 OU. She had the Thr377Met variant as expected but does not have glaucoma at this time. The father of A126 was not available for examination. The seven individuals with normal cup to disc ratios and IOPs ranged in age from 45 to 90 years old. No signs of exfoliation were found in the 15 individuals with the Thr377Met variant examined at the University of Thessaloniki. The incidence of the Thr377Met *MYOC* variant in the glaucoma patients was 59% (13/22).

**Table 2:** Clinical findings in villagers with the Thr377Met *MYOC* variant.

**Code number**	**Age**	**IOP OD/OS**	**CCT OD/OS**	**C/D OD/OS**	**Visual field loss**	**Status**	**MYOC variation**
A33	52	17/18	0.531/.534	0.45/0.45	yes	POAG	Thr377Met
A14	60	18/18	0.488/.491	0.6/0.5	yes	POAG	Thr377Met
A15	82	42/24	0.502/502	0.9/0.9	Yes	POAG	Thr377Met
A16	56	19/27	0.554/.567	0.6/0.65	yes	POAG	Thr377Met
A58	55	33/28	0.520/.526	0.9/0.8	yes	POAG	Thr377Met
A23	68	65/24	0.573/.587	0.94/0.99	yes	POAG	Thr377Met/ Thr377Met
A6	72	18/19	0.542/.539	0.99/ 0.99	yes	POAG	Thr377Met
A54	74	42/21	0.536/.557	0.9/0.85	yes	POAG	Thr377Met/ Arg76Lys
A34	71	15/15	0.545/.536	0.90/0.75	yes	POAG	Thr377Met
A42	74	8/10	0.533/.545	0.85/0.85	yes	POAG	Thr377Met
A31	71	20/17	0.509/.528	0.6/0.6	yes	POAG	Thr377Met/ Arg76Lys
A126	16	25/25	0.556/.557	0.6/0.45	yes OD	POAG	Thr377Met/ Thr377Met
A107	65	24/25	0.612/.610	0.2/0.2	ND	OHT	Thr377Met
A40	43	22/22	0.565/.601	0.3/0.3	no	OHT	Thr377Met
A25	36	22/20	0.630/.585	0.5/0.4	no	POAG	Thr377Met
A3	72	22/24	0.556/.553	0.5/0.6	yes	Suspect	Thr377Met/ Arg76Lys
A21	90	12/12	0.543/.588	0.2/0.2	ND	Normal	Thr377Met
A50	70	12/13	0.572/.565	0.3/0.3	ND	Normal	Thr377Met
A43	68	21/20	0.553/.528	0.3/0.3	ND	Normal	Thr377Met
A125	51	17/15	0.559/.567	0.3/0.3	ND	Normal	Thr377Met
A41	45	18/19	0.598/.591	0.3/0.3	ND	Normal	Thr377Met

Clinical characteristics of the 21 villagers that were found to have the Thr377Met *MYOC* variant is displayed; 13 of whom have POAG. The abbreviations are ND=Not done; OD=right eye; OS=left eye; and C/D=cup-to-disc ratio.

The Arg76Lys polymorphism was found in 31 of the 123 screened villagers. Three of these individuals were diagnosed with POAG; two of them also had the Thr377Met mutation (see Table 3). The same individual described above with normal cup to disc ratios and IOPs and an abnormal visual field had both the Arg76Lys and the Thr377Met variations. The other 27 subjects had only the Arg76Lys polymorphism with the exception of one individual who also had the Thr438Thr variant. One individual with IOPs of 26 and 20 mmHg in the right and left eye, respectively, was reclassified from normal to OHT. Normal cup to disc ratios and IOPs were found in the remaining 26 individuals whose ages ranged from 17 to 81 years.

**Table 3:** Clinical presentation in individuals with the Arg76Lys *MYOC* variant.

**Code number**	**Age**	**IOP OD/OS**	**CCT OD/OS**	**C/D OD/OS**	**Visual field loss**	**Status**	**MYOC variation**
A31	71	20/17	0.509/.528	0.6/0.6	Yes	POAG	Thr377Met/ Arg76Lys
A54	74	42/21	0.536/.557	0.9 Pale/0.85	Yes	POAG	Thr377Met/ Arg76Lys
A82	71	20/32	0.528/.501	0.65/0.9	Yes	XFG	Arg76Lys
A3	72	22/21	0.556/.553	0.3/0.3	Yes	Suspect	Thr377Met/ Arg76Lys
A49	50	9/11	0.582/.576	0.3/0.3	Not Done	Normal	Arg76Lys
A51	60	13/13	0.617/.609	0.1/0.1	Not Done	Normal	Arg76Lys
A26	68	17/17	0.511/.518	0.3/0.3	Not Done	Normal	Arg76Lys
A27	56	09/10	0.585/.572	0.1/0.2	Not Done	Normal	Arg76Lys
A29	17	21/21	0.606/.579	0.4/0.4–0.5	Not Done	Normal	Arg76Lys
A30	64	09/10	0.527/.520	0.3/0.3	Not Done	Normal	Arg76Lys
A57	39	26/24	0.533/.544	0.4/0.4	No	OHT	Arg76Lys
A60	50	18/14	0.546/.553	0.1/0.1	Not Done	Normal	Arg76Lys
A67	64	20/19	0.530/.528	0.3/0.3	Not Done	Normal	Arg76Lys
A68	52	20/16	0.556/.551	0.2/0.5	Not Done	Normal	Arg76Lys
A63	80	14/16	0.558/.565	0.3/0.2	Not Done	Normal	Arg76Lys
A65	81	11/11	0.486/.489	0.2/0.3	Not Done	Normal	Thr438Thr/ Arg76Lys
A71	63	16/18	0.566/.566	0.3/0.3	Not Done	Normal	Arg76Lys
A79	83	17/15	0.585/.592	0.3/0.3	Not Done	Normal	Arg76Lys
A80	61	17/20	0.583/.538	0.4/0.4	Not Done	Normal	Arg76Lys
A81	34	16/14	0.619/.628	0.3/0.3	Not Done	Normal	Arg76Lys
A84	70	19/17	0.516/.528	0.3/0.3	Not Done	Normal	Arg76Lys
A86	78	15/15	0.587/.600	0.2/0.2	Not Done	Normal	Arg76Lys
A93	34	15/15	0.585/.580	0.35/0.35	Not Done	Normal	Arg76Lys
A98	58	16/15	0.539/.502	0.3/0.3	Not Done	Normal	Arg76Lys
A99	59	18/19	0.542/.559	0.2/0.2	Not Done	Normal	Arg76Lys
A122	31	9/9	0.565/.600	0.3/0.3	Not Done	Normal	Arg76Lys
A105	70	13/14	0.602/.594	0.2/0.2	Not Done	Normal	Arg76Lys
A101	59	14/13	0.552/.557	0.1/0.1	Not Done	Normal	Arg76Lys
A117	54	12/13	0.536/.577	0.1/0.1	Not Done	Normal	Arg76Lys
A124	34	19/18	0.510/.505	0.2/0.2	Not Done	Normal	Arg76Lys
A109	70	13/11	0.567/.569	0.3/0.3	Not Done	Normal	Arg76Lys

Clinical characteristics of the 31 villagers with the nonglaucoma-associated Arg76Lys *MYOC* variant are shown. Two of the three individuals with POAG also have the Thr377Met *MYOC* mutation. The abbreviations are ND=Not done; OD=right eye; OS=left eye; and C/D=cup-to-disc ratio.

Ten villagers who were diagnosed with glaucoma had normal *MYOC* sequences, although one had the Tyr347Tyr polymorphism. This person also had exfoliation. Their ages ranged from 49 to 80 years. The maximum IOP in this group was 36 mmHg. Four individuals with normal *MYOC* sequences were classified as suspects based either upon high IOPs (greater than 22 mmHg) or vertical cup to disc ratios of 0.5–0.6.

Five families were identified with two or more individuals with glaucoma. Four families possessed the Thr377Met variation. In the first family, all three individuals with the Thr377Met mutation have glaucoma including A23 who is homozygous for this variant. His sister, A75, also has POAG but has the Tyr347Tyr polymorphism not the Thr377Met variant (see Figure 1). In the second family (see Figure 2), six individuals have POAG, but only five of these have the Thr377Met mutation. In addition, one individual in the older generation (A50) has the Thr377Met variant but had normal ocular findings when examined at the age of 70. Only one of these families (data not shown) had clean segregation of glaucoma with the Thr377Met mutation. The other three families each had one individual with glaucoma with the normal Thr377Thr *MYOC* sequence. Analysis of the haplotype showed that all of the individuals from Taxiarchis with the Thr377Met *MYOC* variant shared the haplotype previously reported in Greek families (not shown) [11].

## Discussion

This small Greek village appears to have a high prevalence of glaucoma (22 out of 126 individuals) approaching 18%. This was a self-selected study so the findings may be biased by individuals with known glaucoma being more likely to enroll in the study. We are in the process of examining all of the villagers to determine the actual prevalence of glaucoma in this village. The Thr377Met *MYOC* mutation was identified in over half of the glaucoma patients. The incidence of *MYOC* mutations in unselected glaucoma patients reportedly ranges between 3%–5% in other populations [10,19,24]. Higher incidences of *MYOC* mutations are found in juvenile glaucoma and mixed-onset primary open-angle glaucoma families (30%–33%) [14].

The age of the glaucoma patients reflected the previously reported age ranges for *MYOC* mutations. The youngest patient was 16 years old and was homozygous for the Thr377Met mutation. The remaining glaucoma patients ranged in age from 36 to 82 years.

In the previous paper reporting the high incidence of familial glaucoma in this village [9], 14 of the 20 individuals diagnosed with glaucoma were from one family. In this study with the information provided to us by the villagers, we identified five different families with glaucoma. Considering that the Thr377Met *MYOC* variant was segregating through four of these families and all individuals with the variant shared the same haplotype, these four families are probably related at some point farther back in their history. Further genealogical work will be required to identify the common ancestor for these individuals.

A confounding factor in glaucoma is the presence in some patients of exfoliation syndrome, which is characterized by the development of white, dandruff-like flakes on anterior segment structures [25]. In the eastern Mediterranean countries, the prevalence of exfoliation in glaucoma cohorts ranges from 25%–47%. In Greece, Konstas and Allen [5] reported that 87% of glaucoma patients undergoing trabeculectomy had exfoliation. Our finding of only two patients with exfoliation in this glaucoma cohort is noteworthy considering that this disease is extremely common in Greece.

The clinical presentation of the two patients homozygous for the Thr377Met *MYOC* mutation is much more severe than that of the heterozygous subjects. Typically, patients with only one copy of the Thr377Met mutation are diagnosed in their fourth decade with maximum IOPs around 30 mmHg [11]. In this study, one of the homozygotes was diagnosed at the age of 16 with difficult to control IOPs. The second villager, also homozygous for the Thr377Met mutation, had an IOP of 65 mmHg in his right eye at the time of examination with no vision left in that eye. Thus, the major impact of homozygosity for the Thr377Met *MYOC* mutation appears to be extremely high and difficult to control pressures.

Homozygosity of three different *MYOC* variants, Lys423Glu, Gln368 STOP, and Arg46STOP, has previously been reported [26-28]. The Lys423Glu variant in the heterozygous state usually presents with severe glaucoma before the age of 40. Normal ocular findings were found in four siblings who were homozygous for the Lys423Glu variant [26]. Hewitt et al. [27] reported a subject that was homozygous for the Gln368STOP *MYOC* mutation with normal ocular findings at the age of 49. In the last case, a Korean proband, homozygous for the Arg46STOP mutation, presented with a severe glaucoma phenotype while her heterozygous relatives did not exhibit any glaucomatous signs [29]. The frequency of the Arg46STOP variant has been shown to be similar in glaucomatous (2.0%) and matched control cohorts (2.2%), suggesting that this sequence variant is likely to be a neutral variant in the heterozygous state [30]. Our findings show that the Thr377Met *MYOC* homozygous state results in a more severe glaucoma phenotype than the heterozygous condition. This stands in contrast to the Lys423Glu and Gln368STOP homozygotes, which have normal ocular features.

The above findings suggest that the Thr377 position has a unique function in *MYOC*. The Thr377 site in the myocilin protein may be phosphorylated by casein kinase II (CK2) [31,32]. CK2 is a ubiquitous, highly pleiotropic, constitutively active Ser/Thr protein kinase that phosphorylates over 300 protein substrates with an S/T-x-x-D/E motif. The substitution of the Thr377 with Met in the myocilin protein potentially results in loss of phosphorylation of this site. Three different scenarios potentially impacted by CK2 phosphorylation include the stabilization of helix unfolding, inhibition of caspase cleavage, which shares the E/D-x-D recognition motif of CK2, and inhibition of protein–protein adhesion modules [31,33,34]. Future research investigating the properties of mutant myocilin, which replaces threonine at position 377 with methionine, will be required to determine the exact mechanism for the more severe presentation in the homozygote compared to the heterozygote.

Caspase cleavage of the D-I-D motif (residues 378–380) in the Thr377Met homozygote would result in the release of the carboxyl peptide. In the Glu368STOP homozygote, this peptide is absent due to the deletion. In the Lys423Glu homozygote, the Thr377 residue is present and available for phosphorylation and thus, inhibition of cleavage of the carboxyl terminus by caspase occurs. Thus, the presence of the carboxyl peptide in the Thr377Met homozygote but not in the Glu368STOP or Lys423Glu homozygotes may be the factor causing the difference in clinical presentation.

Several groups have examined the expression of the Gln368STOP, Lys423Glu, and Thr377Met myocilin proteins [35,36]. Solubility studies have shown that all three substitutions result in decreased solubility of the protein [36]. Interestingly, the Thr377Met mutant is secreted extracellularly while the Gln368STOP and Lys423Glu mutant proteins are retained intracellularly [35]. The differences in presentation between the Thr377Met, Gln368STOP, and Lys423Glu homozygotes may be due to the specific amino acid change, the position in the protein, or the genetic background of the individual.

The incomplete penetrance of the Thr377Met *MYOC* mutation has been previously reported by several groups [12,16,17]. In Finland, a six-generation family with juvenile open-angle glaucoma (JOAG) and primary open-angle glaucoma (POAG) included 20 individuals heterozygous for the mutation; however, only nine were glaucomatous [12]. In Australia, 31 mutation carriers over the age of 18 years were identified in four POAG pedigrees; 23 of whom had either OHT or POAG [17]. The families in our study also show incomplete penetrance of the Thr377Met *MYOC* mutation, suggesting that this particular variant is more likely to be a susceptibility factor than a major glaucoma gene.

Probably the most intriguing finding in this study is the large number of villagers with the Thr377Met *MYOC* and Arg76Lys variants (17% and 25%, respectively). A higher than expected heterozygote frequency may be explained by genetic drift, increased fertility, or heterozygote selective advantage. The high number of villagers with the Thr377Met *MYOC* mutation may result from the isolation of the village, may reflect that the original founder of this mutation was from this village, or may be due to selective advantage of the heterozygous state. Sharing of mutations and high carrier rates support the premise that the origin of the Thr377Met *MYOC* mutation occurred in Taxiarchis and spread from this village to other regions in Greece and ultimately worldwide.
